# Multiple Plasmid Vectors Mediate the Spread of *fosA3* in Extended-Spectrum-β-Lactamase-Producing *Enterobacterales* Isolates from Retail Vegetables in China

**DOI:** 10.1128/mSphere.00507-20

**Published:** 2020-07-15

**Authors:** Luchao Lv, Xiuyu Huang, Jing Wang, Ying Huang, Xun Gao, Yiyun Liu, Qiaoli Zhou, Qianhui Zhang, Jun Yang, Jian-Ying Guo, Jian-Hua Liu

**Affiliations:** a College of Veterinary Medicine, National Risk Assessment Laboratory for Antimicrobial Resistant of Microorganisms in Animals, Guangdong Provincial Key Laboratory of Veterinary Pharmaceutics Development and Safety Evaluation, South China Agricultural University, Guangzhou, China; b Guangdong Laboratory for Lingnan Modern Agriculture, Guangzhou, China; c Key Laboratory of Prevention and Control of Biological Hazard Factors (Animal Origin) for Agrifood Safety and Quality, Ministry of Agriculture of China, Yangzhou University, Yangzhou, China; JMI Laboratories

**Keywords:** *fosA3*, ESBL-producing *Enterobacterales*, retail vegetable, plasmids

## Abstract

The plasmid-mediated fosfomycin resistance gene *fosA3* has been detected in *Enterobacterales* from various sources but has rarely been reported in vegetables. In this study, the aim was to investigate the prevalence of and, subsequently, to characterize *fosA3*-positive *Enterobacterales* isolates from retail vegetables. Seventeen (7.3%) *fosA3*-carrying strains were identified from 233 extended-spectrum-β-lactamase-producing *Enterobacterales* isolates from vegetables. All 17 isolates, including six Escherichia coli, seven Klebsiella pneumoniae, two Raoultella ornithinolytica, and two Citrobacter freundii isolates, carried *bla*_CTX-M_. S1-nuclease pulsed-field gel electrophoresis (S1-PFGE) and hybridization confirmed that the *fosA3* genes in 16 isolates were located on plasmids ranging in size from ∼40 kb to ∼250 kb, except one located on chromosome of C. freundii. All the *fosA3-*carrying plasmids from 16 *fosA3*-positive isolates were successfully transferred into the recipient bacteria by transformation or conjugation. In agreement with data determined with isolates from food animals, the IncHI2/ST3 and IncN-F33:A-:B-/F33:A-:B plasmids were the main vectors of *fosA3* in E. coli. Additionally, F24:A-:B1, IncFII_K_-IncR, IncFII_S_, IncR, and two untypeable plasmids were found for the first time to be vectors for *fosA3* in *Enterobacterales*. The genetic contexts of *fosA3* in 15 *Enterobacterales* isolates differed due to insertion and/or loss of molecular modules mediated by mobile elements. However, all *fosA3* genes were flanked by IS*26*, as commonly observed in other *fosA3*-carrying plasmids. Here, we report a high rate of detection of *fosA3* genes, mediated by multiple plasmid vectors, in ESBL-producing *Enterobacterales* from retail vegetables. FosA3-producing *Enterobacterales* could be transmitted to the human body by direct contact or consumption of vegetables, which might pose a potential threat to public health.

**IMPORTANCE** This report provides important information on the transmission and epidemiology of *fosA3* among *Enterobacterales* isolates from vegetables. The rate of occurrence of *fosA3* in ESBL-producing *Enterobacterales* from retail vegetables is high, and *fosA3* was found to be carried by diverse plasmids. Some novel genetic contexts of *fosA3* and novel *fosA3*-carrying plasmids, including several plasmid types common in K. pneumoniae, were identified, increasing the number of known transfer vectors for the *fosA3* gene and reflecting the complexity of *fosA3* transmission in *Enterobacterales*. The capture of *fosA3* by the resident plasmid of K. pneumoniae will accelerate the spread of *fosA3* in K. pneumoniae, one of the most pathogenic species in clinical medicine. Considering the clinical importance of fosfomycin, and the fact that vegetables are directly consumed, the fosfomycin resistance genes present a risk of transmission to the human body through the food chain and thus pose a threat to public health.

## Abstract (precis)

This report provides important information on the transmission and epidemiology of *fosA3* among *Enterobacterales* isolates from vegetables. The rate of occurrence of *fosA3* in ESBL-producing *Enterobacterales* from retail vegetables is high, and *fosA3* was found to be carried by diverse plasmids. Some novel genetic contexts of *fosA3* and novel *fosA3*-carrying plasmids, including several plasmid types common in K. pneumoniae, were identified, increasing the number of known transfer vectors for the *fosA3* gene and reflecting the complexity of *fosA3* transmission in *Enterobacterales*. The capture of *fosA3* by the resident plasmid of K. pneumoniae will accelerate the spread of *fosA3* in K. pneumoniae, one of the most pathogenic species in clinical medicine. Considering the clinical importance of fosfomycin, and the fact that vegetables are directly consumed, the fosfomycin resistance genes present a risk of transmission to the human body through the food chain and thus pose a threat to public health.

## INTRODUCTION

The increasing rate of bacterial infections caused by multidrug-resistant (MDR) Gram-negative bacteria, including extended-spectrum-β-lactamase (ESBL)-producing *Enterobacterales* and carbapenem-resistant *Enterobacterales* (CRE), has led to a renewed interest in older antimicrobial agents, such as fosfomycin (1, 2). Fosfomycin has broad-spectrum antimicrobial activity against Gram-negative bacteria and some Gram-positive bacteria and acts by inhibiting bacterial cell wall synthesis. It remains one of the first-line drugs used for the treatment of acute uncomplicated urinary tract infections caused by ESBL-producing *Enterobacterales* (3). In clinical medicine, fosfomycin activity against ESBL-producing Escherichia coli remains high (87.7% or greater) (4–6).

However, in recent years, many reports have described the presence of plasmid-mediated fosfomycin resistance (*fos*) genes in *Enterobacterales* isolates around the world (5–7). The *fosA* gene is the most common of the plasmid-mediated fosfomycin resistance genes in *Enterobacterales*, and 10 *fosA* gene types have been identified (7, 8). Among them, *fosA3* is the most frequently gene type and is disseminated mainly in Asian countries (China, South Korea, and Japan) but is detected in countries outside Asia only sporadically (5–15). The plasmid-borne *fosA3* gene likely originated from the chromosome of Kluyvera georgiana (16) and has been detected in several *Enterobacterales* species such as *Escherichia*, *Klebsiella*, *Salmonella*, *Proteus*, and *Citrobacter* spp. from various sources, including livestock animals, pets, clinical human samples, wild animals, and environmental samples (7, 17), but other sources such as vegetables are rarely analyzed (18).

Vegetables may become contaminated with resistance genes via animal manure fertilization, wastewater irrigation, soil, primary processing, or transport or sale. A particularly important factor is that fresh vegetables are often eaten raw, occasionally without washing, although research has shown that washing does not completely remove bacteria from vegetables (19). Thus, the available evidence suggests that resistance genes in vegetables may pose risks to human health. The aims of this study were to investigate the prevalence of and, subsequently, to characterize plasmid-mediated fosfomycin resistance gene *fosA3* in ESBL-producing *Enterobacterales* isolates from retail vegetables in China.

## RESULTS

### Prevalence of plasmid-mediated fosfomycin resistance genes.

Of the 233 ESBL-producing *Enterobacterales* isolates examined in this study, 17 (7.3%) isolates, including 6 (6/28, 21.4%) Escherichia coli, 7 (7/160, 4.4%) Klebsiella pneumoniae, 2 Raoultella ornithinolytica, and 2 Citrobacter freundii isolates, carried *fosA3* (Table 1). In addition, 157 (157/160, 98.1%) K. pneumoniae isolates harbored a chromosome-encoded *fosA* gene. They were not included in further investigation. Other plasmid-mediated fosfomycin resistance determinants were not identified.

**TABLE 1:** Characteristics of *fosA3*-positive *Enterobacterales* isolates from vegetables, China, 2015 to 2016

Isolate^*a*^	Bacterial species	Origin	MLST	Fosfomycin MIC (mg/liter)	Location	Plasmid size (kb)	Other resistance gene(s)^*b*^	Other resistance phenotype(s)^*c*^	Genetic context	GenBank accession no.
TS53CTX	*R. ornithinolytica*	Lettuce		2,048	IncHI2/ST3	253.02	*bla*_CTX-M-14_, *oqxAB*, *mcr-1*, *floR*	CL/FFC/SXT/TET	H	MF135535
TS48CTX	*R. ornithinolytica*	Lettuce		2,048	IncHI2/ST3	253.02	*bla*_CTX-M-14_, *oqxAB*, *mcr-1*, *floR*	CL/FFC/SXT/NEO/ DOX/TET	H	MF135534
6HS20eCTX	E. coli	Lettuce	ST795	256	IncHI2/ST3	250.83	*bla*_CTX-M-14_, *floR*, *mcr-1*, *oqxAB*	FFC/GEN/NEO/TET/CL	I	MF135536
6BF21eCTX	E. coli	Tomato	ST69	1,024	IncHI2/ST3	∼244	*bla*_CTX-M-14_, *floR*, *oqxAB*, *mcr-1*	APR/NEO/STR/TET/ FFC/CL/SXT	NA^*d*^	NA
TS62CTX	E. coli	Lettuce	ST156	2,048	IncN-F33:A-:B-	114.95	*bla*_CTX-M-55_, *floR*, *rmtB*, *mcr-1*	AMK/GEN/FFC/TET/ CL/SXT/CIP	L	MK079574
6BS21eCTX	E. coli	Lettuce	ST2505	2,048	IncN-F33:A-:B-	∼104	*bla*_CTX-M-55_, *floR, oqxAB*, *mcr-1*	TET/STR/FFC/CL/SXT	NA	NA
6BS17eCTX	E. coli	Lettuce	ST410	2,048	IncN-F33:A-:B1	75.17	*bla*_CTX-M-55_, *floR*	NEO/STR/TET/FFC/ SXT/CIP	J	MK416152
6TC9eCTX	E. coli	Cucumber	ST165	2,048	F24:A-:B1	120.96	*bla*_CTX-M-14_	DOX/GEN/TET	G	MK416151
BC6-3	K. pneumoniae	Cucumber	ST875	2,048	F33:A-:B-	65.16	*bla*_CTX-M-55_	TET	K	MK079570
6YF2CTX	K. pneumoniae	Tomato	New	2,048	IncR-IncFIIk:1	153.20	*bla*_CTX-M-38_	TET	E	MK167989
LC3	K. pneumoniae	Cucumber	ST3558	2,048	IncR-IncFIIk:7	129.41	*bla*_CTX-M-24_	TET	B	MK104259
6BS12CTX	K. pneumoniae	Lettuce	ST307	2,048	IncR-IncFIIk:5	156.09	*bla*_CTX-M-24_	FOX/SXT/TET STR/ DOX/GEN	B	MK167987
TS45CTX	C. freundii	Lettuce		256	IncFIIs	114.63	*bla*_CTX-M-14_, *floR*	FOX/SXT/NEO/DOX/ TET/FFC	F	MK167988
LDH4-2	K. pneumoniae	Bean sprouts	ST3557	2,048	IncR	42.87	*bla*_CTX-M-24_, *floR*	FFC/SXT/STR/TET	A	MK079573
6BF16CTX	K. pneumoniae	Tomato	ST1035	1,024	New	55.18	*bla*_CTX-M-38_	STR/FOX/GEN/TET	C	MK079571
LDL3-2	K. pneumoniae	Bean sprouts	ST1407	2,048	New	60.29	*bla*_CTX-M-38_	TET/SXT	D	MN319465
LDL3-3	C. freundii	Bean sprouts		512	Chromosome		*bla*_CTX-M-24_, *floR*, *bla*_CMY-2_	FOX/FFC/SXT/CIP/ STR/DOX/TET	M	CP047247

a Strains with names highlighted with underlining were transformants, with the E. coli DH5a strain as the recipient. The others were transconjugants, with the E. coli C600 strain (streptomycin resistant; MIC of >2,000 mg/liter) as the recipient.

b Names of genes that were transferred by conjugation or transformation as determined by PCR and sequencing are highlighted with underlining.

c All isolates and transconjugants/transformants were resistant to fosfomycin, ampicillin, and cefotaxime, with the exception that the transconjugant of 6YF2CTX was found to be susceptible to cefotaxime. Resistance phenotypes transferred to the recipient by conjugation are highlighted with underlining. AMK, amikacin; APR, apramycin; CIP, ciprofloxacin; CL, colistin; DOX, doxycycline; FFC, florfenicol; FOX, cefoxitin; GEN, gentamicin; NEO, neomycin; TET, tetracycline; STR, streptomycin; SXT, sulfamethoxazole and trimethoprim.

d NA, not available.

All of the *fosA3*-positive isolates were resistant to fosfomycin, ampicillin, and cefotaxime but susceptible to imipenem (MICs of ≤0.25 mg/liter). Screening for resistance genes confirmed that all *fosA3*-carrying isolates were CTX-M producers (six CTX-M-14, four CTX-M-24, four CTX-M-55, and three CTX-M-38) (Table 1). In addition, 10 isolates contained *floR* and 6 isolates carried *mcr-1*. The six isolates (TS53CTX, TS48CTX, 6HS20eCTX, 6BF21eCTX, TS62CTX, and 6BS21eCTX) carrying *mcr-1* were described in a previous study (18).

### Genotyping and genetic background of *fosA3*-harboring isolates.

Pulsed-field gel electrophoresis (PFGE) was successfully conducted for six E. coli and seven K. pneumoniae isolates. The PFGE results showed that the E. coli and K. pneumoniae isolates have different characteristic PFGE patterns (see Fig. S1 in the supplemental material). The *fosA3*-positive E. coli isolates and the K. pneumoniae isolates also belonged to different multilocus sequence types (MLSTs) (Table 1). The observed genetic diversity indicated that the *fosA3*-carrying isolates were clonally unrelated.

10.1128/mSphere.00507-20.1 FIG S1 XbaI-PFGE dendrogram and detailed information about *fosA3*-positive E. coli (A) and K. pneumoniae (B) isolates. Download FIG S1, TIF file, 1.1 MB. Copyright © 2020 Lv et al. 2020 Lv et al. This content is distributed under the terms of the Creative Commons Attribution 4.0 International license.

### Location of *fosA3* and replicon types of plasmids carrying *fosA3*.

The *fosA3*-carrying plasmids were successfully transferred from donors to recipients by conjugation or transformation, except LDL3-3. S1-nuclease pulsed-field gel electrophoresis (S1-PFGE) and hybridization confirmed that the *fosA3* genes in 16 isolates were located on plasmids ranging in size from ∼40 kb to ∼250 kb (Table 1). However, MinION sequencing confirmed that *fosA3* in C. freundii LDL3-3 was chromosomally located (GenBank accession number CP047247). MICs of fosfomycin for all transconjugants/transformants were >1,024 mg/liter. Cotransfer of *bla*_CTX-M_ occurred in all isolates except 6YF2CTX. The *floR* and *mcr-1* genes were cotransferred with *fosA3* to the recipients from three and four donors, respectively (Table 1). The replicon types of the *fosA3* plasmids were IncHI2/ST3 (*n* = 4), IncN1-F33:A-:B- (*n* = 2), IncN1-F33:A-:B1 (*n* = 1), F33:A-:B- (*n* = 1), IncFII_K_ (*n* = 3), F24:A-:B1 (*n* = 1), and untypeable (*n* = 4).

### Characterization of FosA3-encoding plasmids.

The complete sequences of 14 *fosA3*-bearing plasmids were obtained, with the exception of isolates 6BF21eCTX (IncHI2/ST3), 6BS21eCTX (IncN1-F33:A-:B-), and LDL3-3 (chromosomal) (Table 1).

The backbones of three IncHI2/ST3 plasmids (pHNTS48-1, 253,021 bp; pHNTS53-1, 253,021 bp; pHNHS20EC, 250,827 bp) were highly similar to those of other IncHI2/ST3 plasmids from various sources, such as pA3T (KX421096, chicken, China), pHXY0908 (KM877269, chicken, China), pHNSHP45-2 (KU341381, pig, China), pSDE-SvHI2 (MH287084, pig, Australia), cq9 plasmid unnamed1 (CP031547, pig, China), pEC5207 (KT347600, pig, China), and pLS61394-MCR (CP035916, Homo sapiens, China), but with different arrangements of the multidrug resistance region (MRR) (Fig. S2).

10.1128/mSphere.00507-20.2 FIG S2 Comparisons of IncHI2 plasmids (pHNTS53-1, pHNHS20EC, and pHNTS53-1) carrying *fosA3*. Comparative analysis of different plasmids was conducted by using BLAST Ring Image Generator (BRIG) software. Plasmid pHNHS20EC was used as a reference. Each circle represents a different plasmid as follows (from inner to outer ring): cq9 plasmid unnamed1, pA3T, pEC5207, pHNSHP45-2, pHXY0908, pSDE-SvHI2, pHNTS48-1, pHNTS53-1, pLS61394-MCR, and pHNHS20EC. Mobile elements are labeled with green arrows; antibiotic resistance genes are marked with red arrows; transfer (*trth*) genes are marked with blue arrows on the outermost ring. Download FIG S2, TIF file, 2.4 MB. Copyright © 2020 Lv et al. 2020 Lv et al. This content is distributed under the terms of the Creative Commons Attribution 4.0 International license.

**FIG 1:**
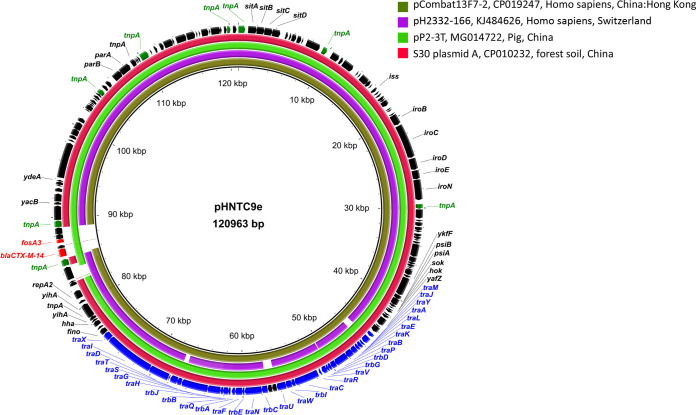
Comparison of F24:A-:B1 plasmid pHNTC9e carrying *fosA3* with other similar plasmids. Each circle represents a different plasmid as follows (from inner to outer ring): pCombat13F7-2, pH 2332-166, pP2-3T, S30 plasmid A, and pHNTC9e. Mobile elements are labeled with green arrows; antibiotic resistance genes are marked with red arrows; transfer (*tra* and *trb*) genes are marked with blue arrows on the outermost ring.

The IncFII type plasmids included one F33:A-:B- plasmid (pHNBC6-3, 65,161 bp), one IncN1-F33:A-:B- plasmid (pHNTS62, 114,949 bp), one IncN1-F33:A-:B1 plasmid (pHNBS17e, 75,167 bp), and one F24:A-:B1 plasmid (pHNTC9e, 120,963 bp). The backbones of plasmids pHNBC6-3 and pHNTS62 shared significant similarity with those of previously reported F33:A-:B-/IncN1-F33:A-:B- plasmids obtained from livestock, dog, animal-derived food, and human in China (Fig. S3a and b). In pHNBS17e, parts of the F33:A-:B- plasmid backbone (the leading region and the conjugal transfer region) were absent (Fig. S3c).The backbone of F24:A-:B1 plasmid pHNTC9e is highly related (99% identity with 97% coverage) to that of pCombat13F7-2 (CP019247), a 151-kb F24:A-:B1 plasmid without *fosA3* isolated from E. coli of human urine in Hong Kong (Fig. 1). The backbone of pHNTC9e was also similar to those of IncHI2/ST3-F24:A-:B1 plasmid pP2-3T (MG014722, E. coli, pig, China) and F24:A-:B1 plasmid A (CP010232, E. coli, soil, China).

10.1128/mSphere.00507-20.3 FIG S3 IncF33 plasmids carrying *fosA3*. (A) Comparison of F33:A-:B- plasmids carrying *fosA3*. pHNBC6-3 was used as a reference. Each circle represents a different plasmid as follows (from inner to outer ring): pHNBC6-3, pHN7A8, pHNMC02, pECB11, pHNMPC43, pHNGD4P177, pHNAH33, and pHNBC6-3 (this study). (B) Comparisons of IncN-F33:A-:B- plasmids carrying *fosA3*. Plasmid pHNTS62 was used as a reference. Each circle represents a different plasmid as follows (from inner to outer ring): pHNFKD271, pHN04NHB3, pHNFP460-1, pEA-1, pE80, pHNAH24, pHNZY32, and pHNTS62 (this study). (C) Genome map of IncN-F33:A-:B1 plasmid pHNBS17e. The blue, black, green, and red parts of the inner ring represent the segments of N/A, F33:A–:B–, IncN, and IncFIB plasmid. Mobile elements are labeled with green arrows; antibiotic resistance genes are marked with red arrows; transfer genes (*tra* and *trb*) are marked with blue arrows on the outermost ring. Download FIG S3, PDF file, 1.5 MB. Copyright © 2020 Lv et al. 2020 Lv et al. This content is distributed under the terms of the Creative Commons Attribution 4.0 International license.

**FIG 2:**
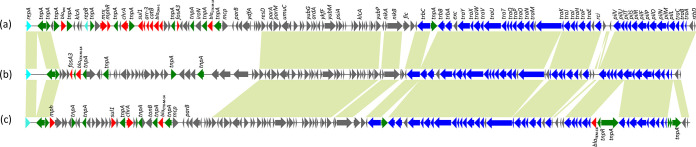
Linear comparisons of complete sequences of IncFII_S_ plasmid pHNTS45-1 with other similar plasmids. (a) p112298-KPC (C. freundii, KP987215, Homo sapiens, China). (b) pHNTS45-1 (pHNTS45-1, MK167988, C. freundii, this study). (c) Strain EN3600 plasmid unnamed3 (CP035635, E. cloacae, Homo sapiens, China). Open reading frames (ORFs) are depicted by arrows. Fluorescent blue, green, red, blue, and gray arrows represent replication genes, mobile elements, resistance genes, plasmid transfer genes, and plasmid backbone genes, respectively. Light green shading denotes regions of shared >90% homology among the different plasmids.

pHNTS45-1, isolated from a C. freundii strain collected from a lettuce sample, was found to be a 114,632-bp IncFII_S_:4-like plasmid. pHNTS45-1 was organized similarly (95% identity with 72% coverage) to p112298-KPC (KP987215) (Fig. 2), a plasmid carrying *bla*_KPC-2_ and *fosA3* that was isolated from a clinical C. freundii isolate in China.

**FIG 3:**
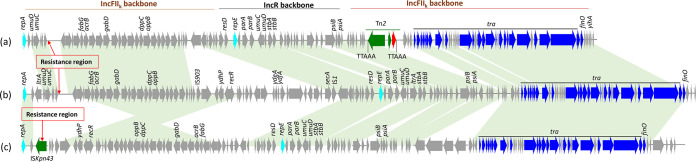
Linear comparisons of complete sequences of IncR-IncFII_k_ plasmids. (a) pHNLC3 (MK104259, K. pneumoniae, cucumber, this study). (b) pHNBS12 (MK167987, K. pneumoniae, lettuce, this study). (c) pHNYF2-1 (MK167989, K. pneumoniae, tomato, this study). Open reading frames (ORFs) are depicted by arrows. Fluorescent blue, green, red, blue, and gray arrows represent replication genes, mobile elements, resistance genes, plasmid transfer genes, and plasmid backbone genes, respectively. Light green shading denotes regions of shared >90% homology among the different plasmids.

**FIG 4:**
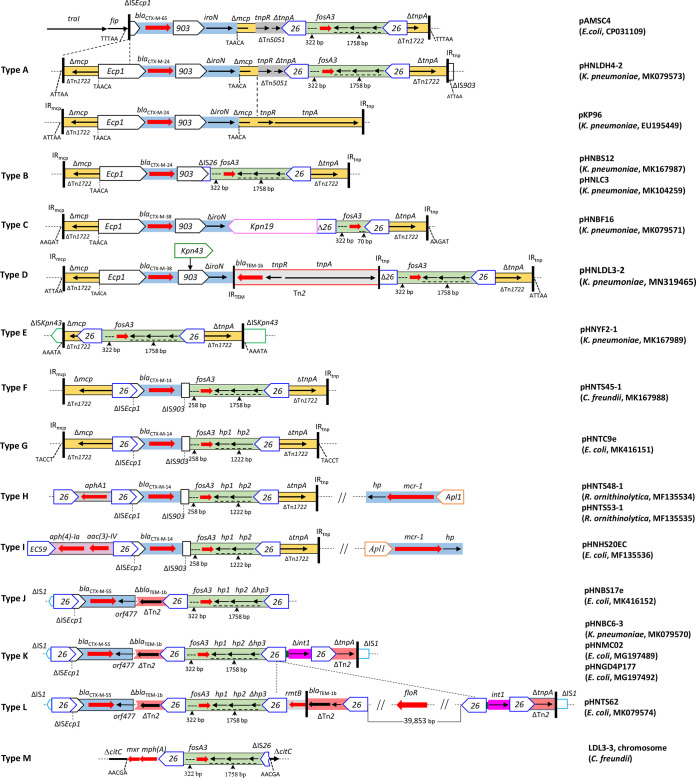
Comparison of the genetic contexts of *fosA3* in this study with others. The extents and directions of orientation of resistance genes (thick red arrow) and other genes are indicated by arrows labeled with gene names. Insertion sequences (ISs) are shown as boxes labeled with the IS name. Labeled vertical arrows with IS boxes denote the insertion position of IS elements. Transposons (Tn) and suspected transposons flanked by direct repeats (DRs) have generally been indicated directly with short dotted lines. Tall bars represent IR of Tn. Horizontal dotted lines are used to represent plasmid backbone.

Three novel *fosA3*-bearing plasmids from K. pneumoniae isolates, pHNBS12 (156,088 bp), pHNLC3 (131,404 bp), and pHNYF2-1 (153,201 bp), were found to be multireplicon plasmids belonging to IncR-FII_K_:5, IncR-FII_K_:7, and IncR-FII_K_:1, respectively. Plasmids pHNBS12, pHNLC3, and pHNYF2-1 had backbones that were similar to each other and were organized similarly (99% identity with 63% coverage) to IncR-IncFII_K_:7 plasmid pKpN06-CTX (CP012993, K. pneumoniae, human, Canada) harboring *bla*_CTX-M-15_. In pHNBS12, the resistance region harboring *fosA3* and *bla*_CTX-M-24_ embedded in an incomplete Tn*1722* was inserted into the IncFII_K_ backbone (Fig. 3; see also Fig. 4). This arrangement was also observed in pHNLC3 with the same insertion site. However, pHNLC3 also had an insertion of Tn*2* containing β-lactamase resistance gene *bla*_TEM-1b_ into the IncFII_K_ backbone with 5 bp of target site duplication (TSD) (TTAAA) (Fig. 3). The resistance region of pHNYF2-1 had only one resistance gene (*fosA3*) embedded in Tn*1722* which was inserted in the IS*Kpn43* element with a 5-bp TSD (AAATA) (Fig. 3; see also Fig. 4).

**FIG 5:**
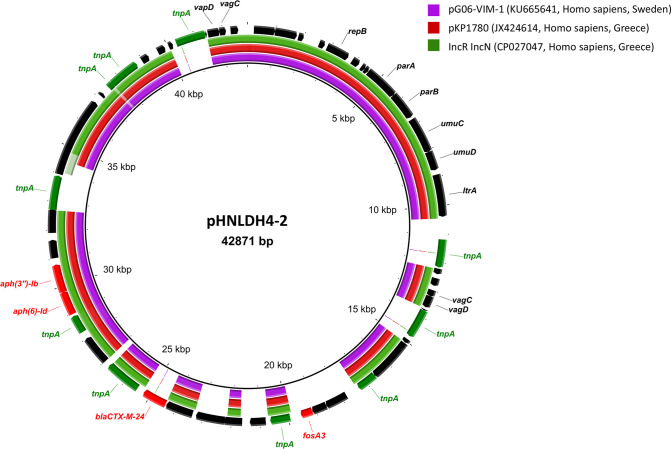
Comparisons of plasmids related to pHNLDH4-2 (IncR). Each circle represents a different plasmid as follows (from inner to outer ring): pG06-VIM-1, pKP1780, and IncR IncN. Mobile elements are labeled with green arrows; antibiotic resistance genes are marked with red arrows on the outermost ring.

Plasmid pHNLDH4-2 was 42,871 bp in length. It comprised a 21,903-bp MRR and 20,968 bp IncR plasmid backbone, including core genes coding for plasmid replication (*repB*), a plasmid partition system (*parA* and *parB*), a toxin-antitoxin system (*vagCD*), and an SOS mutagenesis system (*umuCD*) (Fig. 5). BLAST searches indicated the high similarity (99% identity with 71% coverage) of pHNLDH4-2 to *bla*_VIM_-carrying plasmids pG06-VIM-1 (KU665641, K. pneumoniae) and pKP1780 (JX424614, K. pneumoniae). IncR plasmids were previously reported to be associated with the transmission of *bla*_KPC_, *bla*_VIM_, and *bla*_NDM_ among *Enterobacterales* (20). However, no reports of *fosA3*-harboring IncR plasmid have been found so far. Although IncR-F33:A-:B- plasmids are increasingly reported as vectors of *fosA3*, *bla*_CTX-M_, *bla*_KPC_, *rmtB*, and other resistance genes in K. pneumoniae, and in hypervirulent K. pneumoniae in China in particular, the *fosA3* gene likely originated from F33:A-:B- plasmid pHN7A8 or a pHN7A8-like plasmid (21–23).

**FIG 6:**
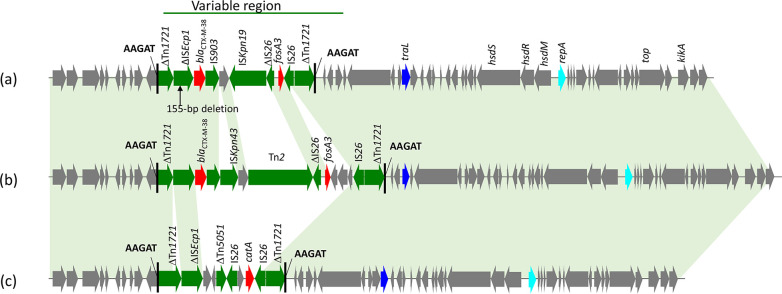
Linear comparisons of complete sequences of untypeable plasmids pHNBF16 and pHNLDL3-2. (a) pHNBF16 (MK079571, K. pneumoniae, tomato, this study). (b) pHNLDL3-2 (MN319465, K. pneumoniae, bean sprouts, this study). (c) pX39-3 (CP023980, K. variicola, Homo sapiens, China). Open reading frames (ORFs) are depicted by arrows. Fluorescent blue, green, red, blue, and gray arrows represent replication genes, mobile elements, resistance genes, plasmid transfer genes, and plasmid backbone genes, respectively. Light green shading denotes regions of shared >90% homology among the different plasmids.

pHNBF16 (55,179 bp) and pHNLDL3-2 (60,297 bp) were (untypeable) novel *fosA3*-bearing plasmids from K. pneumoniae isolates. BLAST comparisons showed that pHNBF16 and pHNLDL3-2 possessed backbone organizations (99% identical, 80% to 86% coverage) similar to that of plasmid pX39-3 (CP023980) from a clinical Klebsiella variicola isolate in China but shared less than 30% coverage with other plasmids (Fig. 6). All three plasmids contained an essential replication gene (*repB*), several genes of type IV secretion systems, and one mobile resistance module. The mobile resistance module in three plasmids was an incomplete Tn*1722* embedded with several resistance genes (e.g., *fosA3*, *bla*_CTX-M_, and *catA2*) and was inserted in the plasmid backbone at the same location, generating a 5-bp TSD (AAGAT) (Fig. 6).

### The *fosA3*-containing genetic structures.

Twelve genetic structures were identified in the 14 sequenced plasmids and designated type A to type L (Fig. 4), with types B, C, and D representing novel genetic contexts. In 11 structures from 13 plasmids, the *bla*_CTX-M_ gene was located upstream of the *fosA3* gene. Among the 14 sequenced plasmids, 9 harbored *fosA3* genes that were flanked by two IS*26* elements oriented in the same direction, but an incomplete IS*903* was found to be located upstream of *fosA3* in the remaining 5. Three different lengths of spacer regions (1,758 bp, 1,222 bp, and 70 bp) were identified between the 3′ ends of *fosA3* and IS*26* (Fig. 4).

As shown in Fig. 4, type A consists of a mosaic structure bounded by two fragments of Tn*1722* which interrupted IS*903* with a 5-bp TSD (ATTAA) in plasmid pHNLDH4-2. The *fosA3* gene was embedded in a typical transposition unit (IS*26*-*fosA3*-*hp1*-*hp2*-Δ*hp3*-IS*26*) which truncated *tnpA* of Tn*1722*. The *mcp* gene of Tn*1722* was interrupted by the very common genetic structure of *bla*_CTX-M-9G_ (IS*Ecp1*-*bla*_CTX-M-24_-IS*903D*-*iroN*), flanked by a 5-bp TSD (TAACA), which was also observed in K. pneumoniae plasmid pKP96 (EU195449). The 779-bp segment of transposon Tn*5051* was found to be located between the *fosA3* resistance module and a 687-bp fragment of Tn*1722* (Δ*mcp-*150 bp). The type A structure was similar to the corresponding region of plasmid pAMSC4 (CP031109) found in E. coli isolated from Giant panda in China. Compared with pHNLDH4-2, ΔTn*1722* and part of IS*Ecp1* was absent, *bla*_CTX-M-65_ was associated with a genetic structure (*traI*-*fip*-ΔIS*Ecp1*-*bla*_CTX-M-9G_-IS*903D*-*iroN*) commonly observed in IncN plasmids, and a 779-bp-shorter Δ*tnpA* gene corresponding to Tn*1722* adjacent to the *fosA3* module was present in pAMSC4.

The *fosA3* resistance regions of pHNBS12 and pHNLC3 in the type B genetic structure were similar to those of type A. However, IS*26* (26-bp remnant) located upstream of *fosA3* was truncated by IS*903*, which may explain the deletion of a 3,425-bp segment (*iroN*-Δ*mcp*-ΔTn*5051*) in the type B structure (Fig. 4). A 308-bp-longer (1,517 bp) Δ*tnpA* gene of Tn*1722* was observed in type B. The lack of TSD flanking ΔTn*1722* in type B suggests that this may have occurred by homologous recombination rather than transposition.

The *fosA3* module (ΔIS*26*-322 bp-*fosA3*-18 bp-IS*26*) in the type C (pHNBF16) genetic structure distinctly differed from structures observed in other plasmids; only a 14-bp remnant of *hp1* was found downstream of *fosA3* in pHNBF16 (Fig. 4). The IS*26* element upstream of *fosA3* was truncated at the 3′ end by IS*Kpn19*, which also truncated the *bla*_CTX-M-38_ transposition unit (IS*Ecp1*-*bla*_CTX-M-38_-IS*903D*-*iroN*), leading to deletion of 319 bp of the 3′ end of *iroN* in pHNBF16 (Fig. 4).

The genetic environment of type D (pHNLDL3-2) was also related to those of type A to type C. The *bla*_CTX-M_ transposition unit (IS*Ecp1*-*bla*_CTX-M-38_-ΔIS*903*-Δ*iroN*) was inserted in Tn*1722*, except that IS*903D* was interrupted by IS*Kpn43* at an 18-bp right inverted repeat (IRR) and *iroN* was truncated by a complete Tn*2* transposon carrying β-lactam resistance gene *bla*_TEM-1b_ (Fig. 4). Similarly to type A and B, *fosA3* was associated with the typical transposition unit (IS*26*-322 bp-*fosA3*-1758 bp-IS*26*), except the upstream IS*26* (610 bp) was truncated by Tn*2*. Although Tn*1722* in pHNLDL3-2 was flanked by a 5-bp TSD (ATTAA) as seen in type A, the Tn*1722* downstream of the *fosA3* module was identical to that in the type B structure (Fig. 4).

The genetic context of *fosA3* in pHNYF2-1 (type E) was a typical *fosA3* transposition unit (IS*26*-*fosA3*-*hp1*-*hp2*-Δ*hp3*-IS*26*) inserted in Tn*1722* as observed in the above plasmids. The separating Tn*1722* had a 784-bp-shorter Δ*mcp* gene and a 1,517-bp Δ*tnpA* gene (observed in type B structures) and was flanked by a 5-bp TSD (AAATA) (Fig. 4). The resistance regions of pHNTS45-1 (type F) and pHNTC9e (type G) were highly similar, except for the loss of a 536-bp segment between *fosA3* and the downstream IS*26* in pHNTC9e, and 5-bp TSD was not observed in pHNTS45-1. In both cases, ΔIS*903*, instead of IS*26*, was located upstream of *fosA3* with a 258-bp spacer; and the upstream *bla*_CTX-M_ transposition unit (ΔIS*Ecp1*-*bla*_CTX-M-14_-ΔIS*903*) was truncated by IS*26* at IS*Ecp1*. Both plasmids had a Δ*mcp* gene that was 472 bp longer than the equivalent gene seen in types A to D and a 1,517-bp Δ*tnpA* gene (as seen in type B). The genetic structure (IS*26*-ΔIS*Ecp1*-*bla*_CTX-M-14_-ΔIS*903*-258 bp-*fosA3*-1,222 bp-IS*26*-ΔTn*1722*) in pHNTC9e (type G) was also identified in IncHI2/ST3 plasmids pHNTS48-1 (type H), pHNTS53-1 (type H), and pHNHS20EC (type I) (Fig. 4).

The type K genetic structure in pHNBC6-3 (F33:A-:B-) showed 99% nucleotide identity to that found in our previously described pHNMC02 F33:A-:B- plasmid (MG197489, chicken, E. coli) and pHNGD4P177 (MG197492, pig, E. coli) (24) and differed by only three nucleotide changes. The resistance region was bound by fragments of ΔIS*1* and contained four IS*26* elements flanking three different segments associated with a *fosA3* resistance module (Fig. 4). The first segment comprised the genetic fragment of IS*Ecp1*-*bla*_CTX-M-55_-*orf477*-Δ*bla*_TEM-1b_ which is flanked by IS*26* oriented in opposite directions. The second segment contained a typical *fosA3* module (322-bp *fosA3*-*hp1*-*hp2*-Δ*hp3*) flanked by two copies of IS*26*. The two fragments described above constituted the resistance region of pHNBS17e (type J). The last segment contained parts of a 5′ conserved segment (5′-CS), 3′-CS, one copy of IS*26*, and an incomplete Tn*2*.

Plasmid pHNTS62 (type L) showed a variable region related to the type K genetic structure. It had an ∼3.7-kb-longer fragment containing *rmtB*, an incomplete Tn*2*, and one IS*26* element, adjacent to the *fosA3* resistance region (Fig. 4). This type might have evolved from type K through multiple recombination events between IS*26* elements.

In addition, one C. freundii isolate, LDL3-3, carried *fosA3* on a chromosome with a genetic context (type M) different from those of the types mentioned above. In this type, macrolide resistance gene *mph*(A) and *fosA3* module (IS26-322 bp-*fosA3*-1758 bp-ΔIS26) were inserted into a chromosome gene (*citC*) with a 5-bp TSD (AACGA) (Fig. 4).

## DISCUSSION

In our study, *fosA3* was found to be responsible for fosfomycin resistance in ESBL-producing *Enterobacterales* strains from vegetables. *fosA3* has been detected in many countries around the world but mainly occurs in E. coli from food animals in China (5, 10, 24–27). However, there are very few related reports on vegetable sources, with only one related study on *fosA3* (1.2%) in ESBL-producing E. coli isolates in Germany reported recently (13). In this study, we found that the rate of detection of *fosA3* was relatively high (7.3%) in ESBL-producing *Enterobacterales* from vegetables, especially in E. coli (21.4%). The widely distributed FosA3-producing E. coli strain in food animals in China (10, 24–27) might contribute to an equivalently high rate of prevalence of *fosA3* in vegetable isolates, considering that excrement from farm animals carrying the resistant bacteria can contaminate vegetables through irrigation and other means.

We found that the high prevalence of *fosA3* was not due to clone spread but was instead due to horizontal transfer through plasmids. Additionally, plasmid types carrying *fosA3* were abundant, and over eight types of plasmids have been shown to mediate the spread of *fosA3* in *Enterobacterales* from vegetables. Some plasmid types, such as IncHI2/ST3 and IncN-F33:A-:B-/F33:A-:B-, which were previously reported to be the main vectors mediating the spread of *fosA3* in E. coli from food animals in China (24–27), showed high sequence similarity with those found in other sources, especially livestock, suggesting that they may have come directly from food animals. However, other plasmid types, such as F24:A-:B1, IncR-IncFII_K_, IncFII_S_, IncR, and two untypeable plasmids, were the first to be identified as carriers of *fosA3*. IncFII_K_ plasmids are common in K. pneumoniae, where they are found to be responsible for the acquisition and dissemination of resistance genes, but are rare in other *Enterobacterales* species (28). In this study, the three IncR-IncFII_K_ plasmids were also found to be carried by K. pneumoniae. The capture of *fosA3* by the resident plasmid of K. pneumoniae will accelerate the spread of *fosA3* in K. pneumoniae, one of the most pathogenic species in clinical medicine.

Although the plasmids carrying *fosA3* in this study are distinct, *fosA3* is mostly associated with IS*26* and is also frequently associated with *bla*_CTX-M_, in accordance with previous studies (9, 10, 24–27, 29). IS*26* plays an important role in disseminating multiple-resistance determinants in Gram-negative bacteria (20). IS*26* is able to move via replicative transposition and self-targeted transposition. Moreover, IS*26*-mediated transposition is effective and may explain the rapid dissemination of *fosA3* (30). Novel genetic contexts (types B, C, and D) were also identified in this study, and these might be formed through insertions, rearrangements, and/or deletions mediated by mobile elements such as IS*26* and IS*903* (24). The diversity of plasmid types and genetic contexts reflects the complexity of *fosA3* transmission in *Enterobacterales*.

In summary, this study revealed a high rate of detection of *fosA3* in ESBL-producing *Enterobacterales* from vegetables, where *fosA3* was carried by diverse plasmids. Some novel *fosA3*-carrying plasmids were identified, increasing the number of known transfer vectors for *fosA3*. IS*26* may be responsible for mediating the dissemination of *fosA3* among various plasmids and *Enterobacterales* species. Considering the clinical importance of fosfomycin, and the fact that vegetables are directly consumed, the fosfomycin resistance genes represent a risk of transmission to the human body through the food chain and thus pose a threat to public health. It is therefore necessary that further monitoring of fosfomycin resistance in vegetables be performed along with strong relevant investigations.

## MATERIALS AND METHODS

### Sampling.

From May 2014 to August 2016, a total of 1,340 vegetable samples (300 g/sample), including 317 cucumber, 273 lettuce, 258 tomatoes, 256 carrot, and 236 bean sprout samples, were randomly collected from 20 farmers markets and 15 supermarkets located in five districts (Baiyun, Tianhe, Haizhu, Yuexiu, and Liwan) of Guangzhou. Samples were collected using a sterile sampling bag and were transported to the laboratory in a cold box within 4 h. Sampling date, sampling location, and other data were recorded.

### Bacterial recovery and identification and detection of antimicrobial resistance genes.

The samples were washed with sterilized deionized water. The middle parts of cucumber, tomato, and carrot samples, the leaf part of lettuce samples, and the cotyledon part of bean sprout samples were selected for further treatment. Each sample was cut into small sections of 0.1 to 0.5 cm. Then, about 2 to 3 g of sample was placed into 4 ml sterilized LB broth medium for enrichment cultivation at 37°C for approximately 16 to 18 h. Bacterial liquid was incubated on CHROMagar ESBL medium (CHROMagar Microbiology, Paris, France) to select ESBL-producing *Enterobacterales*. The colonies suspected the be ESBL producers were selected and identified by matrix-assisted laser desorption ionization–time of flight mass spectrometry (MALDI-TOF MS) (Shimadzu, Japan), and some were confirmed by 16S rRNA sequencing. Only a single isolate of the same species was isolated from each vegetable sample. A total of 233 ESBL-producing *Enterobacterales* isolates, including K. pneumoniae (*n* = 160), E. coli (*n* = 28), Enterobacter cloacae (*n* = 15), C. freundii (*n* = 7), R. ornithinolytica (*n* = 3), and others (*n* = 20), were recovered from vegetable samples (lettuce samples, *n* = 80; cucumber samples, *n* = 41; carrot samples, *n* = 54; bean sprout samples, *n* = 25; tomato samples, *n* = 33). All 233 isolates were screened for the presence of fosfomycin resistance genes using PCR and DNA sequencing (primers are listed in Table S1 in the supplemental material). The *fosA3*-positive strains were also evaluated for the presence of *bla*_CTX−M_ (CTX-M-1 and CTX-M-9 groups), *bla*_CMY-2_, *rmtB*, and *floR* using PCR (primers are listed in Table S1). The genotype of *bla*_CTX−M_ was confirmed by DNA sequencing.

10.1128/mSphere.00507-20.4 TABLE S1 Primers and probes used in the study. Download Table S1, DOC file, 0.04 MB. Copyright © 2020 Lv et al. 2020 Lv et al. This content is distributed under the terms of the Creative Commons Attribution 4.0 International license.

### Antimicrobial susceptibility testing.

Antimicrobial susceptibility testing was performed by the agar dilution method or broth microdilution method, and the results were interpreted according to Clinical and Laboratory Standards Institute (CLSI) criteria (31). The antimicrobial drugs tested included fosfomycin, ampicillin, cefotaxime, cefoxitin, imipenem, tetracycline, doxycycline, gentamicin, amikacin, streptomycin, apramycin, neomycin, florfenicol, ciprofloxacin, colistin, and sulfamethoxazole-trimethoprim. For determining the MIC of fosfomycin, agar was supplemented with glucose-6-phosphate (G-6-P) (25 mg/liter). E. coli ATCC 25922 was used as the control strain.

### Molecular typing.

All *fosA3*-positive isolates were subjected to pulsed-field gel electrophoresis (PFGE) after digestion with the XbaI restriction enzyme. PFGE patterns were analyzed with BioNumerics software (Applied Maths, Sint-Martens-Latem, Belgium). Isolates harboring the *fosA3* gene were also subjected to MLST analysis, which was performed according to the guidelines provided at the MLST database website (https://pubmlst.org/).

### Conjugation and transformation assays.

The location of *fosA3* was determined by S1 nuclease digestion and hybridization with *fosA3*. Conjugation experiments were carried out by broth mating using a streptomycin-resistant (MIC, >2,000 mg/liter) E. coli C600 strain as the recipient. Transconjugants were selected on MacConkey agar supplemented with 2,000 mg/liter streptomycin and 400 mg/liter fosfomycin. Transformation experiments were carried out in those cases where the conjugation experiments failed. Antimicrobial susceptibility testing was conducted on transconjugants and transformants. The transfer of the resistance genes (*fosA3*, *bla*_CTX-M_, *mcr-1*, and *floR*) was confirmed by PCR. The replicon types of the *fosA3*-carrying plasmids were determined using the protocol provided in the Plasmid MLST database (http://pubmlst.org/plasmid/).

### Whole-genome sequencing and plasmid sequencing.

Whole genomic DNAs of nine FosA3-producing *Enterobacterales* strains (6BS21eCTX, TS62CTX, BC6-3, 6YF2CTX, LC3, 6BS12CTX, LDH4-2, LDL3-2, and LDL3-3) were extracted and were fully sequenced using a HiSeq platform (Illumina, San Diego, CA, USA). The *fosA3*-carrying plasmids were extracted from transconjugants/transformants using a Qiagen plasmid midi kit (Qiagen, Valencia, CA, USA). The plasmids were sequenced with two different platforms, i.e., an Illumina HiSeq platform and a Oxford Nanopore Technologies (ONT) MinION platform. Hybrid assemblies were implemented using Unicycler version 0.4.4. A rapid barcoding sequencing kit was used to construct the libraries sequenced in a MinION device as previously reported (32).

### Bioinformatics analysis.

Open reading frames (ORFs) were predicted using ORF Finder (https://www.ncbi.nlm.nih.gov/gorf/gorf.html), and annotation was performed using the RAST server (http://rast.nmpdr.org/), ISfinder (https://www-is.biotoul.fr/), Plasmidfinder (https://cge.cbs.dtu.dk/services/PlasmidFinder/), ResFinder (https://cge.cbs.dtu.dk//services/ResFinder/), BLAST (http://blast.ncbi.nlm.nih.gov/Blast.cgi), and Gene Construction kit 4.0 (TextcoBio Software, Inc., Raleigh, NC, USA). Subsequent analysis was performed using DNASTAR (Lasergene, Inc., Madison, WI, USA), RAST (33), and Easyfig version 2.1 (34).

### Accession number(s).

The complete sequences of *fosA3*-carrying plasmids and chromosome have been deposited in GenBank under accession numbers MK079570, MK079571, MK079573, MK079574, MK104259, MF135534, MF135535, MF135536, MK167987, MK167988, MK167989, MK416151, MK416152, MN319465, and CP047247.
