# Supplementary figures and images for: Multiple Plasmid Vectors Mediate the Spread of fosA3 in Extended-Spectrum-β-Lactamase-Producing Enterobacterales Isolates from Retail Vegetables in China
Source: mSphere. 2020 Jul 15;5(4):e00507-20. doi: 10.1128/mSphere.00507-20 (PMC7364219; doi:10.1128/mSphere.00507-20)

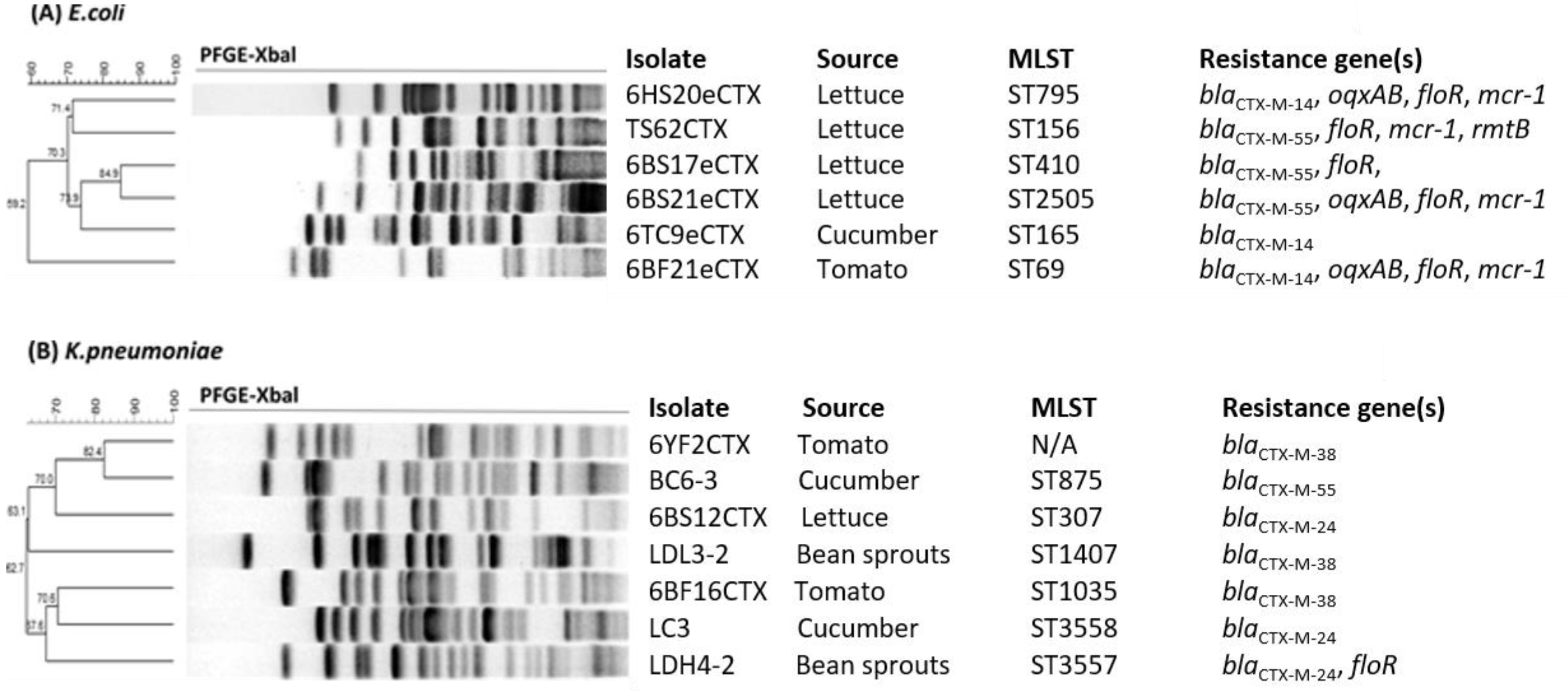

Supplement: FIG S1 [file mSphere.00507-20-sf001.tif]

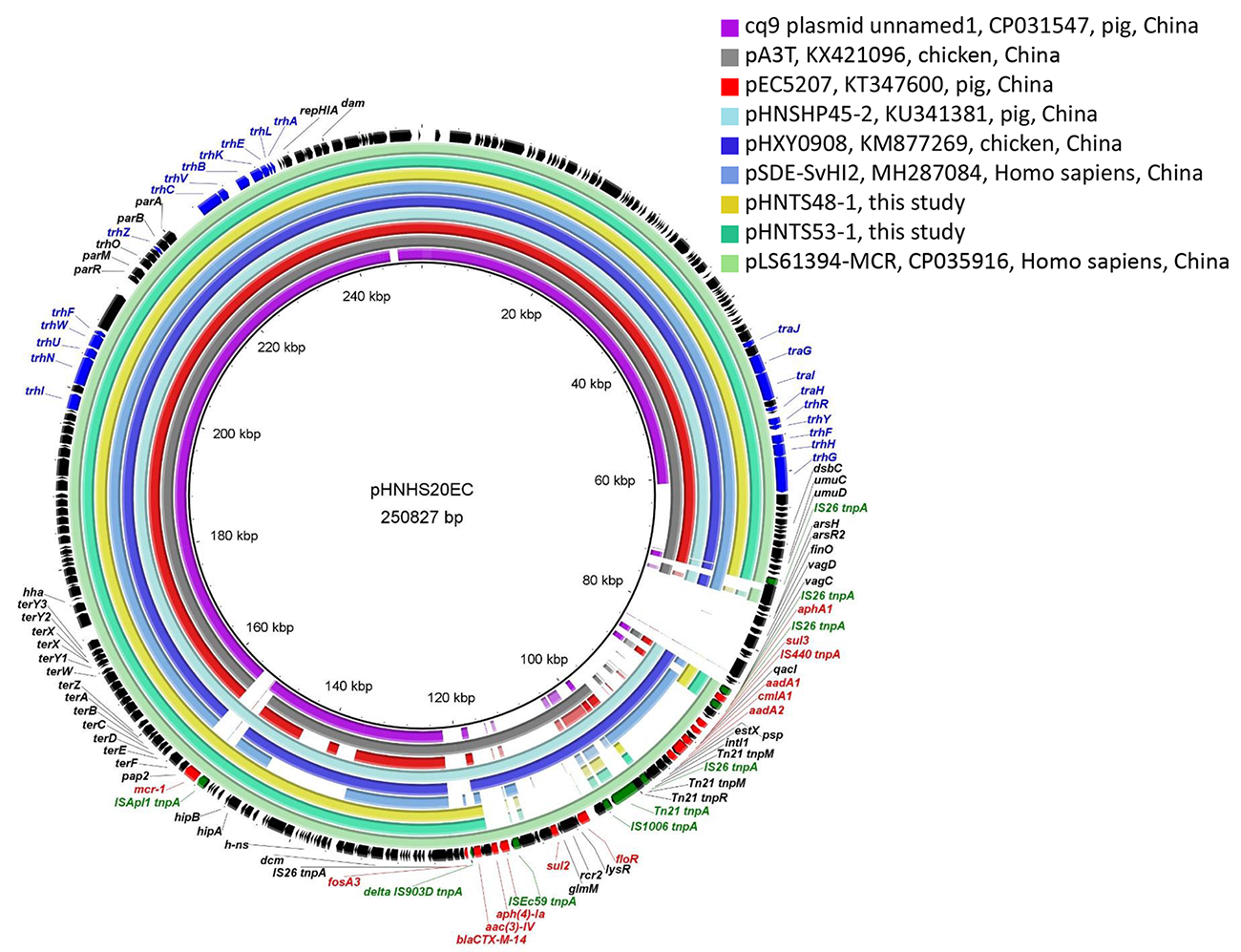

Supplement: FIG S2 [file mSphere.00507-20-sf002.tif]

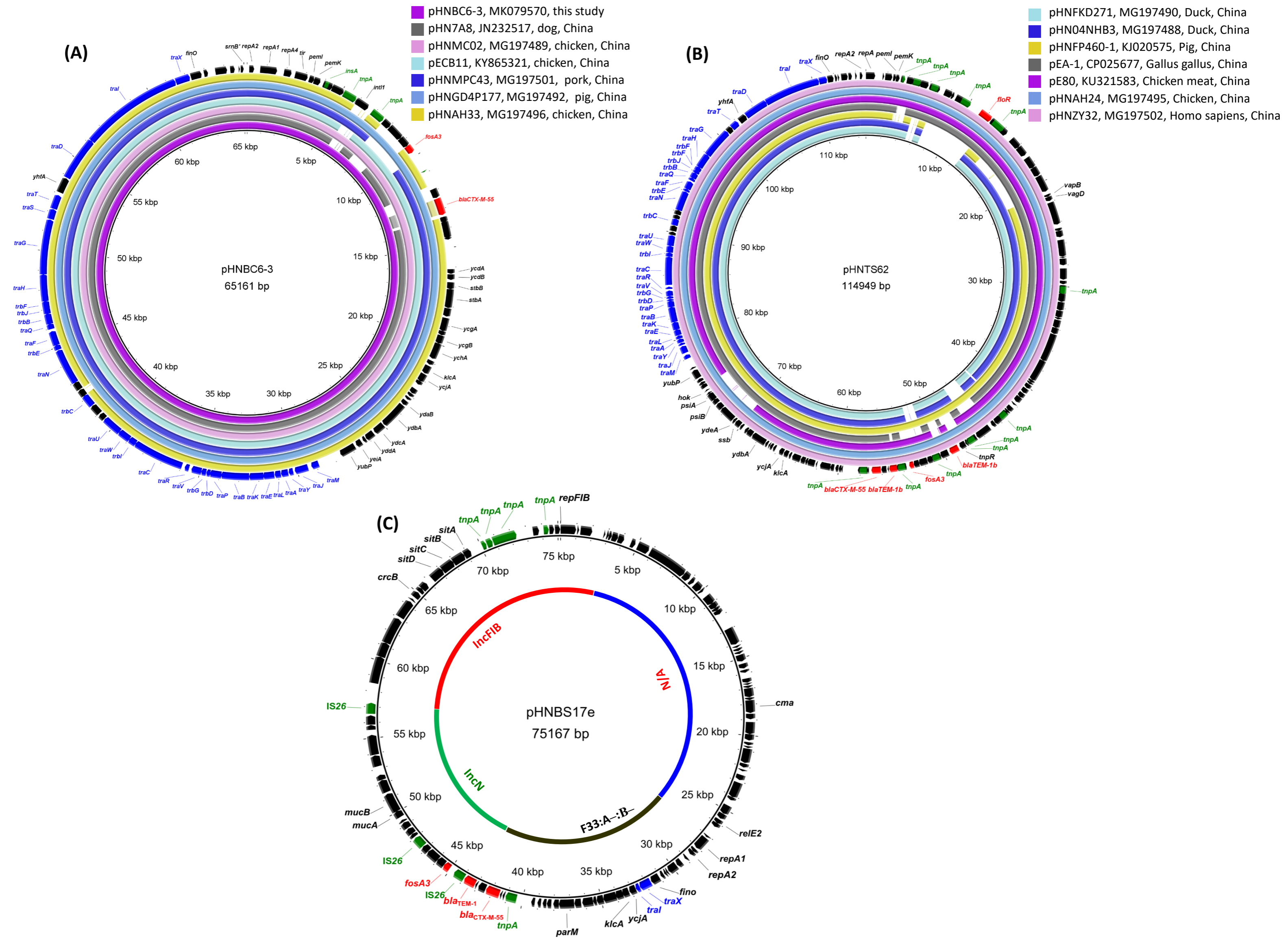

Supplement: FIG S3 [file mSphere.00507-20-sf003.pdf]
