# Supplementary material for: Multiple Plasmid Vectors Mediate the Spread of fosA3 in Extended-Spectrum-β-Lactamase-Producing Enterobacterales Isolates from Retail Vegetables in China
Source: mSphere. 2020 Jul 15;5(4):e00507-20. doi: 10.1128/mSphere.00507-20 (PMC7364219; doi:10.1128/mSphere.00507-20)
Supplement: TABLE S1 [file mSphere.00507-20-st001.doc]

**TABLE S1** Primers and probes used in the study

| **Oligonucleotides** | **Sequence (5'→3')** | **Size(bp)** | **Annealing temp (°C)** |
| --- | --- | --- | --- |
| *fosA6*-like-F | ATGCTGAGTGGACTGAATC | 228 | 52.0 |
| *fosA6*-like-R | ATCGGCTTCGCTAATACTAA |
|  |  |  |  |
| *fosA*-F  *fosA*-R  *fosA2*-F  *fosA2*-R  *fosA3*-F  *fosA3*-R  *fosA4*-F  *fosA4*-R  *fosA5*-F  *fosA5*-R  *fosA6*-F  *fosA6*-R  *fosA7*-F  *fosA7*-R  *fosC2*-F  *fosC2*-R  CTX-M-1-F  CTX-M-1-R  CTX-M-9-F  CTX-M-9-R  *rmtB*-F  *rmtB*-R  *mcr-1*-F  *mcr-1*-R  *floR*-F  *floR*-R | ATCTGTGGGTCTGCCTGTCGT  ATGCCCGCATAGGGCTTCT  GCAATCACTCAACCATCTGACC  TGAAGACCATTCCGGCATAGG  GCGTCAAGCCTGGCATTT  GCCGTCAGGGTCGAGAAA  CTGGCGTTTTATCAGCGGTT  CTTCGCTGCGGTTGTCTTT  TATTAGCGAAGCCGATTTTGCT  CCCCTTATACGGCTGCTCG  CCGGCTTTGAGATGCTGATG  TGAAGGTGGCGATGGAGTG  TCTGAACCACTTAACGCTTGC  CCCGAAACGCATTCCAGAG  TGGAGGCTACTTGGATTTG  AGGCTACCGCTATGGATTT  CTTCCAGAATAAGGAATCCC  CGTCTAAGGCGATAAACAAA  TGACCGTATTGGGAGTTTG  ACCAGTTACAGCCCTTCG  ACATCAACGATGCCCTCAC  AAGTTCTGTTCCGATGGTC  CGGTCAGTCCGTTTGTTC  CTTGGTCGGTCTGTAGGG  CTGAGGGTGTCGTCATCTAC  GTCCCGACAATGCTGACTAT | 271  404  282  230  177  611  400  209  949  902  725  309  673 | 59.5  55.7  57.5  60.0  55.0  55.1  54.8  50.0  56.9  56.8  52.0  52.5  56.0 |
